# Potential Confounding of Particulate Matter on the Short-Term Association between Ozone and Mortality in Multisite Time-Series Studies

**DOI:** 10.1289/ehp.10108

**Published:** 2007-08-02

**Authors:** Michelle L. Bell, Jee Young Kim, Francesca Dominici

**Affiliations:** 1 School of Forestry and Environmental Studies, Yale University, New Haven, Connecticut, USA; 2 National Center for Environmental Assessment, U.S. Environmental Protection Agency, Research Triangle Park, North Carolina, USA; 3 Department of Biostatistics, Johns Hopkins Bloomberg School of Public Health, Baltimore, Maryland, USA

**Keywords:** confounding, mortality, ozone, particulate matter, PM_10_, PM_2.5_, sensitivity analysis

## Abstract

**Background:**

A critical question regarding the association between short-term exposure to ozone and mortality is the extent to which this relationship is confounded by ambient exposure to particles.

**Objectives:**

We investigated whether particulate matter < 10 and < 2.5 μm in aerodynamic diameter (PM_10_ and PM_2.5_) is a confounder of the ozone and mortality association using data for 98 U.S. urban communities from 1987 to 2000.

**Methods:**

We *a*) estimated correlations between daily ozone and daily PM concentrations stratified by ozone or PM levels; *b*) included PM as a covariate in time-series models; and *c*) included PM as a covariate as in *d*), but within a subset approach considering only days with ozone below a specified value.

**Results:**

Analysis was hindered by data availability. In the 93 communities with PM_10_ data, only 25.0% of study days had data on both ozone and PM_10_. In the 91 communities with PM_2.5_ data, only 9.2% of days in the study period had data on ozone and PM_2.5_. Neither PM measure was highly correlated with ozone at any level of ozone or PM. National and community-specific effect estimates of the short-term effects of ozone on mortality were robust to inclusion of PM_10_ or PM_2.5_ in time-series models. The robustness remains even at low ozone levels (< 10 ppb) using a subset approach.

**Conclusions:**

Results provide evidence that neither PM_10_ nor PM_2.5_ is a likely confounder of observed ozone and mortality relationships. Further investigation is needed to investigate potential confounding of the short-term effects of ozone on mortality by PM chemical composition.

Previous research demonstrated an association between short-term exposure to ozone and increased risk of mortality using time-series analyses of 95 U.S. urban communities (Bell et al. 2004), and similar associations have been demonstrated by other recent multicity studies (Gryparis et al. 2004) and meta-analyses (Anderson et al. 2004; Bell et al. 2005; Ito et al. 2005; Levy et al. 2005; Thurston and Ito 2001). A key issue regarding interpretation of these and similar studies is potential confounding by particulate matter (PM) pollution. PM is also associated with increased risk of mortality for short-term exposure (Katsouyanni et al. 1997; Samet et al. 2000a) and could co-vary with ozone levels due to parallel sources of particles and ozone precursors (e.g., transportation).

An earlier analysis of 95 U.S. urban communities found that the overall national relative rate estimate for the relationship between ozone exposure over the previous week and nonaccidental mortality was robust to adjustment of PM with an aerodynamic diameter < 10 μm (PM_10_) (Bell et al. 2004). Further, the community-specific relative rates were robust to PM_10_ adjustment. More recently, a data set of these communities plus three additional communities was used to investigate the exposure–response curve and potential threshold effects for the mortality and ozone relationship (Bell et al. 2006). Results from multiple methods provided strong evidence that if a threshold exists, it is below current regulatory standards and nearing natural background concentrations. None of the statistical models that assumed a nonlinear relationship between ozone and mortality provided significant improvement over the traditional log-linear model. However, this analysis did not include consideration of PM as a confounder. Although the 95-community study (Bell et al. 2004) provides evidence against the theory that PM_10_ confounds the observed associations between ozone and mortality, critical questions remain, such as whether *a*) confounding occurs by particles with an aerodynamic diameter < 2.5 μm (PM_2.5_); *b*) the degree of confounding varies in correspondence to different ozone or PM levels; and *c*) confounding occurs differently by season. Hence, additional research on the degree to which PM may confound ozone and health relationships is needed and is the topic of this study.

Analysis of confounding by PM for studies on the health effects of ozone is hindered by data availability. Ozone is typically measured daily, but often only for the warm season (e.g., April–October). PM data are collected every 3–6 days for the entire year, generally. Further, routine measurements of PM_2.5_ in the United States did not begin until 1999. Therefore, analysis on the short-term health effects of ozone adjusted by PM usually necessitates a smaller sample size than research of either pollutant alone.

Our purpose in the present paper is to explore whether results from ozone and mortality time-series studies are robust to sensitivity analysis regarding potential confounding by PM, using more rigorous analysis than has been previously applied. We investigated PM metrics of PM_10_ and PM_2.5_. Previous work on this issue has focused primarily on PM_10_, although strong mortality associations have been demonstrated for the fine fraction (Kan et al. 2007; Ostro et al. 2006; Schwartz et al. 1996). Because joint analysis of ozone and PM decreases the sample size, we apply several techniques to examine confounding, beyond the traditional inclusion of the confounder as a covariate in the time-series models. For PM to be a confounder for ozone and mortality relationships, concentrations of PM must co-vary with ozone levels. We estimated the correlations between PM (PM_10_ and PM_2.5_) and ozone levels for various strata of long-term average ozone and PM concentrations. We also investigated whether adjustment by PM affects results from threshold analysis, which examines whether there exists an association between ozone and mortality at low levels of ozone.

## Methods

This study considers daily weather, pollution, and mortality data for 98 U.S. urban communities over a 14-year period (1987 to 2000). Communities are defined as a single county or set of contiguous counties. A list of the communities is provided elsewhere (Bell et al. 2006). Daily weather data for temperature and dew point temperature for each community were originally obtained from the National Climatic Data Center. Daily levels of ozone, PM_10_, and PM_2.5_ were obtained from the U.S. Environmental Protection Agency (EPA) Aerometric Information Retrieval Service, now called the Air Quality System database (2007). We used measurements from multiple monitors within an urban community to represent community-wide exposure for pollution or weather variables. The daily number of nonaccidental deaths in each community was originally obtained from the National Center for Health Statistics (Hyattsville, MD).

These publicly available data sources have been processed to generate community-wide estimates for pollution and weather variables for the National Morbidity, Mortality, and Air Pollution Study (NMMAPS) (Dominici et al. 2005; Samet et al. 2000b, 2000c). Additional information on the processing of weather and pollution data, including how values from multiple monitors were combined to generate county-level averages, is available in previous reports (Samet et al. 2000b).

We applied time-series analysis to estimate the relationship between the log of the expected daily mortality rate in each community and the average of the same and previous day’s 24-hr ozone levels (lag


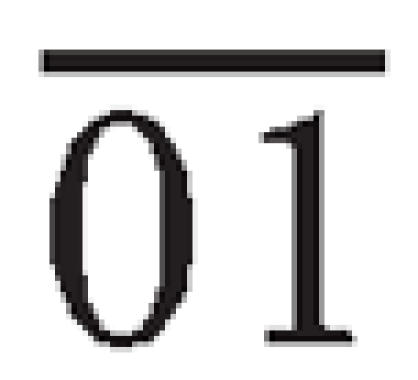


). Previous analysis showed that these days of exposure have higher effect estimates for ozone’s relationship with mortality than other days (Bell et al. 2004). Variables were included for day of the week and for natural cubic splines of temperature, dew point temperature, adjusted previous day’s temperature, adjusted previous day’s dew point temperature, and a variable representing time to adjust for long-term trends and seasonality. The adjusted weather variables include a natural cubic spline of the average of the 3 previous days’ temperature and an analogous variable for dew point temperature, adjusted to avoid correlation with same day temperature and dew point temperature (Bell et al. 2004; Samet et al. 2000b). Community-specific estimates were then combined to generate an overall national estimate, accounting for the statistical uncertainty of each community’s estimate, using Bayesian hierarchical modeling.

We explored potential confounding by PM through several approaches. First, we estimated the correlation between daily 24-hr average ozone and PM levels, for PM_10_ and PM_2.5_, within strata of days with different ozone concentrations. Specifically, we calculated the correlation between same-day ozone and PM levels for days within each of the following strata of ozone concentrations: < 10, 10–20, 20–40, 40–60, 60–80, and > 80 ppb. We estimated correlation coefficients for the actual data and for seasonally detrended data based on a 91-day moving average as follows:


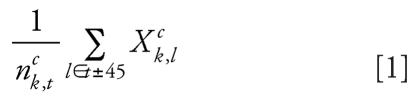


equals the 91-day moving average of the concentration of pollutant *k* for community *c* centered at time *t*;


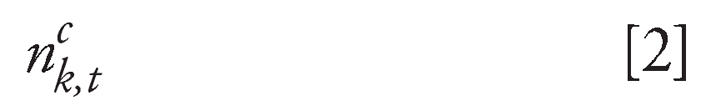


equals the number of days with observations for pollutant *k* for community *c* for a 91-day moving average centered at time *t*.

We conducted sensitivity analysis by estimating the correlation between ozone and PM within various ozone strata for: *a*) different ozone metrics, using the daily 8-hr maximum and daily 1-hr maximum, rather than the 24-hr average ozone values; *b*) the lag structures used in the time-series models, as the lag


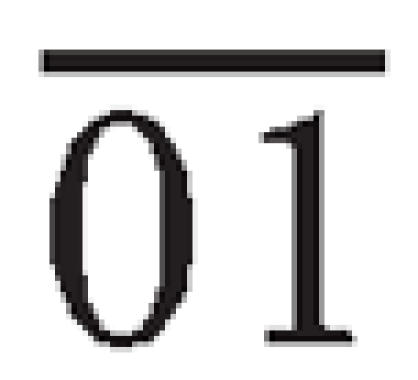


ozone and lag 1 PM, rather than same-day ozone and PM; and *c*) by season and region. Seasons were defined as three month periods (e.g., summer as June–August) and the seven geographic regions were previously defined in NMMAPS (Industrial Midwest, Northeast, Northwest, Southern California, Southeast, Southwest, and Urban Midwest) (Peng et al. 2005).

We also calculated the correlation between ozone and PM for days within specified levels of PM_10_ or PM_2.5_. If PM and ozone co-vary within a given level of pollutant (e.g., high PM concentrations), PM may be a potential confounder for those time periods. Such a condition would imply that PM is a more likely confounder for some seasons or communities than others, based on pollution levels. We investigated the relationship between ozone and PM at various strata to explore whether confounding is possible within different concentration levels.

Second, we considered inclusion of PM_10_ or PM_2.5_ as a covariate in the community-specific time-series models. A lag of 1 day was used for PM variables because this lag structure provided the most significant and largest effect in previous studies (Peng et al. 2005). For comparability, the same data set was used for models with and without adjustment for PM. For example, results from models including adjustment by PM_10_ at lag 1 day were compared with results from models without PM_10_ adjustment, but including only days with PM_10_ lag 1 data available. We considered the robustness of the national effect estimate and the effect estimates for individual communities to inclusion of PM in the time-series models.

We applied a subset approach, which fits the time-series model for a subset of the days with ozone levels below a specified value, *s.* We considered *s* values from 10 to 60 ppb at 10-ppb increments to investigate whether PM acts as a confounder at lower levels of ozone pollution. Results from the subset approach were investigated for robustness to inclusion of PM_10_ at lag 1 day as a covariate. Adjustment by PM_2.5_ was not applied for the subset approach due to sample size constraints. The subset approach was developed to investigate threshold effects. If a threshold value exists, effect estimates for ozone and mortality would be expected to be null for *s* values below the threshold. These model forms, including the community-specific time-series model and the subset approach, are described further elsewhere (Bell et al. 2004, 2006).

## Results

### Frequency of data

Ozone data were available for 81.3% of days over the study period (1987–2000) on average across the 98 communities. Forty-three of the communities measured ozone only during the warm season (e.g., April–October or March–November) for at least some years. PM_10_ data was measured in 93 communities, and PM_10_ data were available for 30.4% of the study days on average across those 93 communities. For PM_2.5_, data were available for 11.1% of the study days on average across the 91 communities that measured PM_2.5_. However, if PM and ozone are included into a regression model simultaneously, even less information is present. Only 25.0% of study days had data on both ozone and PM_10_, on average across the 93 communities with PM_10_ data. Only 9.2% of study days had both PM_2.5_ and ozone data on average across the 91 communities that measured PM_2.5_. These percent-ages are for the actual days with data available, not the days included in various subsequent analyses, which will vary slightly depending on the pollutants used in analysis and the lag structure.

### Correlation between PM and ozone, stratified by pollution levels, season, and region

**Figure 1:**
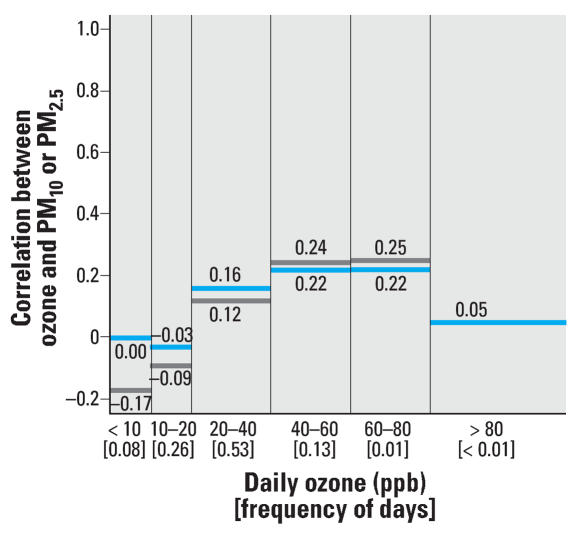
Frequency of daily ozone concentrations (between brackets) and correlation between same-day ozone and PM_10_ (blue) and PM_2.5_ (gray) levels within specified strata of ozone concentrations. Not enough data were available for days with ozone levels > 80 ppb to generate a representative correlation coefficient.

Preliminary evidence as to whether daily levels of PM might confound the short-term effects of ozone on mortality can be gained by calculating the correlation between day-to-day variations in ozone and PM and by exploring whether these correlations vary with respect to the ozone concentrations. Figure 1 shows three features of ozone data within specified strata of ozone levels: *a*) the frequency of ozone days (shown on the *x*-axis); *b*) the Pearson correlation between daily 24-hr average ozone and PM_10_ levels for 93 communities; and *c*) the Pearson correlation between daily 24-hr average ozone and PM_2.5_ levels for 91 communities. For example, 26% of the days have ozone values of between 10 and 20 ppb, and the correlation between PM_10_ and ozone for this stratum of ozone is −0.03. Figure 1 indicates a lack of correlation between ozone and PM_10_ or PM_2.5_ levels at different strata of ozone concentrations. The Pearson correlation coefficients ranged from < 0.00 to 0.22 for correlation within various strata of PM_10_ and −0.17 to 0.25 for correlation within various strata of PM_2.5_

We also calculated the correlation coefficients between same-day ozone and PM_10_ and PM_2.5_ using seasonally detrended data for all pollutants, within ozone strata corresponding to those listed above minus the overall mean ozone level using the 91-day moving average described in “Methods” (results not shown). The correlation coefficients between detrended PM_2.5_ and ozone within each strata ranged from 0.02 to 0.21, whereas the correlation coefficients between detrended PM_10_ and ozone ranged from 0.05 to 0.19.

We estimated the correlation between daily levels of ozone and PM for various levels of PM concentrations (results not shown). Specifically, for strata defined as days with PM_10_ values < 10, 10–20, 20–30, 30–40, 40–50, and > 50 μg/m^3^, the correlation coefficient between ozone and PM_10_ in each strata ranged from −0.08 to 0.10. With PM_2.5_ strata of < 5, 5–10, 10–15, 15–20, 20–25, and > 25 μg/m^3^, the correlation coefficient between ozone and PM_10_ in each strata ranged from 0.03 to 0.10. This indicates the lack of a correlation between PM and ozone at various levels of PM.

**Figure 2:**
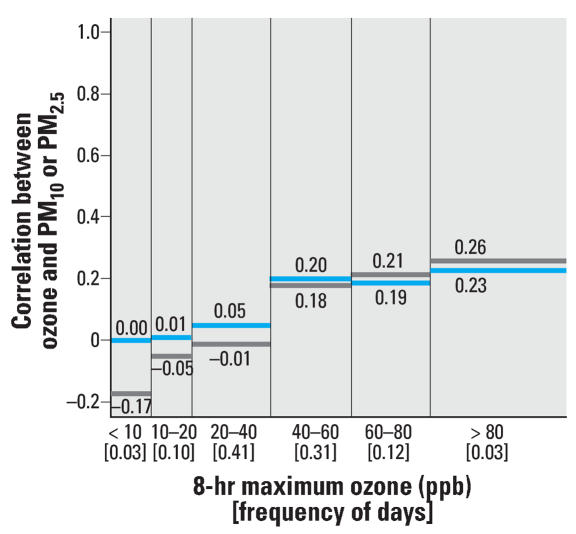
Frequency of the daily 8-hr maximum ozone concentrations (between brackets) and correlation between same day ozone and PM_10_ (blue) and PM_2.5_ (gray) levels within specified strata of ozone concentrations

**Figure 3:**
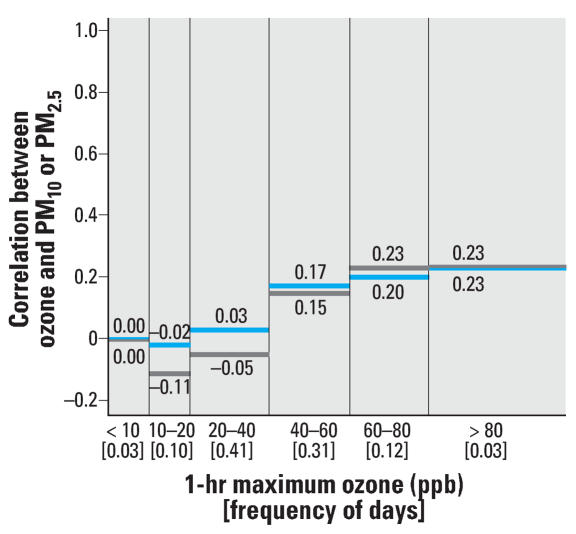
Frequency of the daily 1-hr maximum ozone concentrations (between brackets) and correlation between same day ozone and PM_10_ (blue) and PM_2.5_ (gray) levels within specified strata of ozone concentrations.

**Figure 4:**
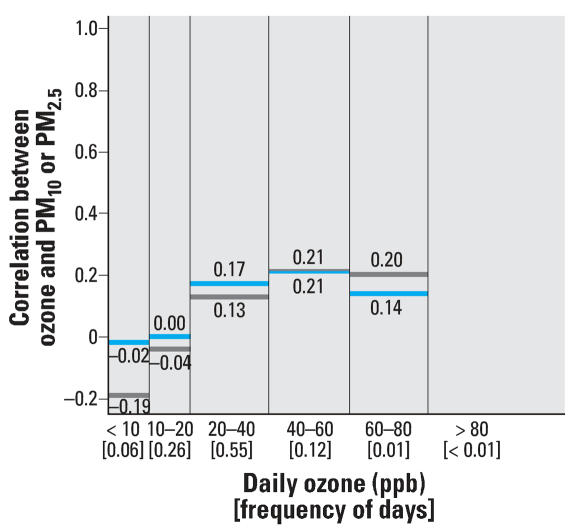
Frequency of the average of the same and previous day’s ozone concentrations (between brackets) and correlation between ozone and the previous day’s PM_10_ (blue) and the previous day’s PM_2.5_ (gray) levels within specified strata of ozone concentrations. Not enough data were available for days with ozone levels > 80 ppb to generate a representative correlation coefficient.

We performed sensitivity analyses with respect to the correlation analysis at various ozone levels. Whereas the original analysis considered same-day pollution levels using daily ozone levels, we also considered the daily 8-hr maximum and the daily 1-hr maximum ozone values, as shown in Figures 2 and 3, respectively. Results based on the lag structures used in the time-series modeling (i.e., average of the same and previous day’s ozone levels and the previous day’s PM levels), are provided in Figure 4. These findings are similar to those in the original analysis based on same-day data using 24-hr ozone levels.

We examined the relationship between same day 24-hr PM and ozone levels by season and region as shown in Table 1. On a national basis, no season demonstrated strong correlations between either PM metric and ozone levels. Highest correlations were observed in spring and fall, and lowest in winter for both PM metrics, for the national data. The correlations between ozone and particles varied by region, with the highest correlations in the Industrial Midwest and Northeast regions for PM_10_ and in the Industrial Midwest and Southeast for PM_2.5_.

### Inclusion of PM as a covariate in time-series models

**Figure 5:**
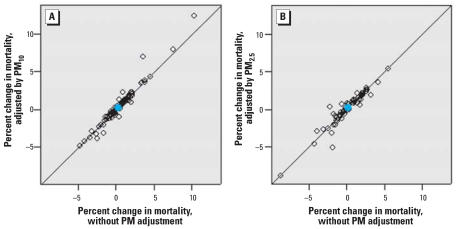
Percent increase in daily nonaccidental mortality per 10-ppb increase in the average of the same and previous day’s ozone levels (lag 
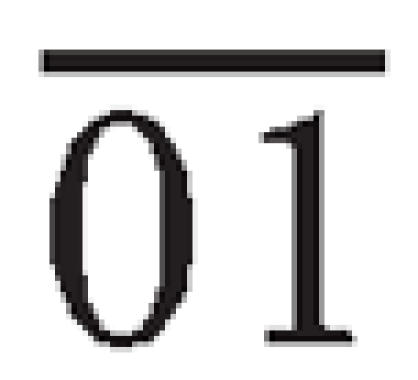
), with and without adjustment by PM_10_ (*A*) and PM_2.5_ (*B*) at lag 1 day. Open symbols represent community-specific estimates; blue circles represent overall national effects.

Previous analyses showed that community-specific and national average estimates of the short-term effects of ozone exposure over the previous week on mortality were robust to adjustment by PM_10_ at a lag of 1 day (Bell et al. 2004). We extended this analysis to include three additional communities, considered ozone exposure for the average of the same and previous day’s ozone levels, and also considered adjustment by PM_2.5_ at a 1-day lag. Although 91 communities had data for PM_2.5_, only 62 had sufficient data for both ozone and PM_2.5_ for the community-specific time-series analysis. Figure 5 shows the percent increase in nonaccidental mortality per 10-ppb increase in the lag


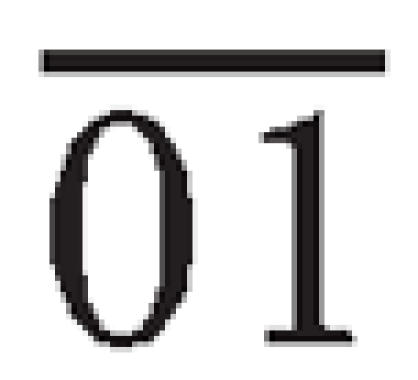


ozone with and without adjustment for PM_10_ (Figure 5A) and PM_2.5_ (Figure 5B) at lag 1. The national relative rate and individual community relative rate estimates are robust to inclusion of either PM metric as a covariate.

Table 2 provides the national effect estimates with and without adjustment by PM_10_ and PM_2.5_. These results demonstrate that although the overall national effect estimates are robust to PM adjustment, the size of the 95% posterior intervals increases dramatically with such analysis, and statistical significance is lost. This is likely attributed to the greatly decreased sample size.

### Subset approach with time-series models

**Figure 6:**
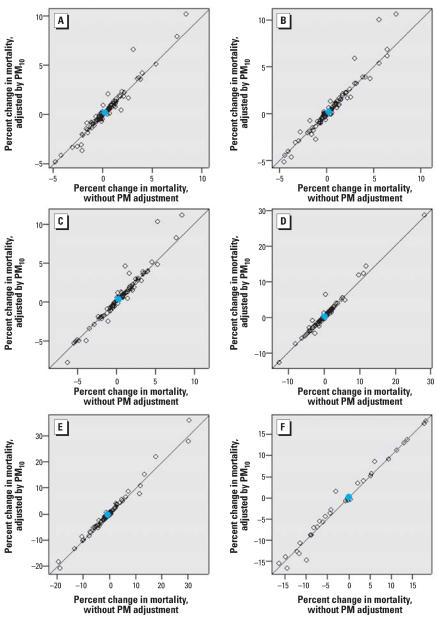
Percent increase in daily nonaccidental mortality per 10 ppb increase in the lag 
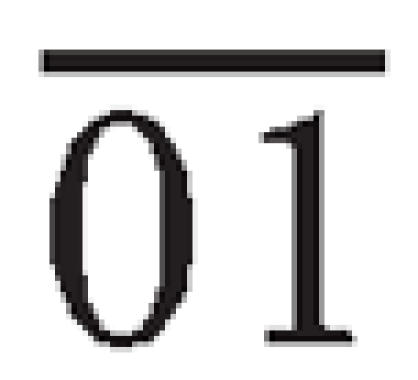
 ozone levels, with and without adjustment by PM_10_ at lag 1 day, using the subset approach. (*A*) 93 communities, *s* = 60; (*B*) 93 communities, *s* = 50; (*C*) 93 communities, *s* = 40; (*D*) 91 communities, *s* = 30; (*E*) 83 communities, *s* = 20; (*F*) 34 communities, *s* = 10. The analysis without adjustment by PM includes only days for which lag 1 PM_10_ data are available. Only days with ozone data < *s* are included. Open symbols represent community-specific estimates; blue circles represent overall national effects.

We applied the subset approach with adjustment by PM_10_ at a lag of 1 day. Figure 6 compares community-specific and national effect estimates at varying levels of *s* for the subset approach, with and without adjustment for PM_10_. For example, Figure 6A applies the time-series model, considering only days with ozone levels less than the *s* value of 60 ppb. Figure 5A is analogous to a subset approach with an *s* of ∞. Of the 98 communities, 93 had PM_10_ data; however, the number of communities decreases as *s* is lowered, because some communities do not have sufficient data for model convergence and in some cases no data at low ozone concentrations. Results indicate that community-specific and national effect estimates are robust to adjustment by PM_10_, for various values of *s*, including when only days with ozone levels < 10 ppb are considered.

## Discussion

An important issue in the interpretation of health risks associated with exposure to ozone is the assessment of potential confounding by co-pollutants, particularly PM. Until more data become available through continued monitoring, especially for PM_2.5_, population-based studies using ambient air pollution data as a surrogate for community-level exposure are severely limited by sample size for analysis of the pollutants jointly. This is particularly problematic for studies with a short time frame of data or for single-city studies. However, with a long time frame, such as our 14-year data set, analysis is still possible. This study examined the robustness of the association between short-term exposure to ozone and risk of mortality to adjustment for PM_10_ and PM_2.5_ using several methods. Initial correlation analyses indicated that, on average across all communities, ozone levels were not highly correlated to PM_10_ or PM_2.5_ levels within strata of ozone concentrations. The Pearson correlation coefficients ranged from < 0.00 to 0.22 for correlation with PM_10_ and −0.17 to 0.25 for correlation with PM_2.5_, and correlations were similarly low using seasonally detrended data, other lag structures, or other ozone exposure metrics. For days with low ozone concentrations (daily ozone < 20 ppb), the correlations were virtually zero for PM_10_ and negative for PM_2.5_.

Several previous time-series analyses suggest lack of evidence for confounding by PM_10_ or total suspended particles (TSP) including the United States and Europe, as well as single-city studies in Latin America and Korea (see discussion in U.S. EPA 2006). This work also provides evidence that the relationship between short-term exposure to ozone and mortality is not confounded by adjustment for PM_10_ or PM_2.5_, at any strata of ozone or PM concentrations.

Our results further show that the national effect estimate and community-specific relative rates of the short-term effects of ozone on mortality are robust to adjustment for PM_2.5_, based on the 62 U.S. communities with PM_2.5_ data available. Previously, only a limited number of studies have examined potential confounding by PM_2.5_ for ozone and mortality. A study of seven Pennsylvania and New Jersey counties from May 1992 to September 1995 found that peak ozone and mortality effect estimates were robust to adjustment by PM_2.5_ and PM_10_ (Lipfert et al. 2000). In a meta-analysis of previously conducted time-series studies of ozone and mortality, Levy et al. (2005) found that effect estimates for ozone and mortality were slightly higher in communities with higher annual or summer correlations between PM_2.5_ and ozone levels; however, the relationship was not statistically significant.

A remaining question regarding PM as a potential confounder for ozone is the differential effect of PM based on chemical composition. This work uses total mass from PM based on specified size distributions, but does not account for the different particle mixtures. For instance sulfate PM_2.5_ contributes a greater fraction of total mass to PM_2.5_ in the eastern United States, whereas nitrate plays a larger role in the western United States. Effect estimates for PM_2.5_ and hospital admissions show temporal and spatial patterns (Domenici et al. 2006), with the largest effects in the Northeast in summer (Peng et al. 2005), which could be the result of differential toxicity of particles. As additional data on PM_2.5_ chemical characterization become available, analysis of confounding by different chemical components or set of components should be investigated.

In summary, we find that PM is unlikely to confound the ozone and mortality relationship. This conclusion is drawn from the correlation analysis between ozone and PM_10_ or PM_2.5_, direct adjustment by PM_10_ and PM_2.5_ in time-series models, and direct adjustment by PM_10_ for days with ozone levels less than a specified cutoff *s*. Although data limitations restrict analysis of PM and ozone simultaneously, collectively our results support the hypothesis that PM does not confound the observed ozone and mortality associations.

## Figures and Tables

**Table 1:** Correlations between same day ozone and PM levels, by season and region.

	No. of communities	Winter	Spring	Summer	Fall	Yearly
PM_10_						
Industrial Midwest	19	0.37	0.44	0.44	0.39	0.41
Northeast	15	0.34	0.44	0.36	0.44	0.40
Urban Midwest	6	0.24	0.25	0.22	0.26	0.24
Southwest	9	0.00	0.02	−0.02	0.10	0.03
Northwest	11	−0.17	−0.20	−0.13	−0.11	−0.16
Southern California	7	0.19	0.08	0.12	0.19	0.14
Southeast	25	0.33	0.35	0.31	0.31	0.32
United States	93	0.23	0.26	0.24	0.26	0.25
PM_2.5_						
Industrial Midwest	19	0.18	0.39	0.43	0.44	0.36
Northeast	13	0.05	0.26	0.16	0.43	0.25
Urban Midwest	4	0.22	0.31	0.15	0.32	0.20
Southwest	9	−0.15	−0.08	−0.17	−0.15	−0.14
Northwest	11	−0.32	−0.34	−0.39	−0.24	−0.31
Southern California	7	−0.25	−0.22	−0.25	−0.15	−0.23
Southeast	26	0.38	0.47	0.30	0.37	0.39
United States	90	0.09	0.21	0.12	0.22	0.16

**Table 2:** Percent increase in mortality per 10 ppb increase in lag 
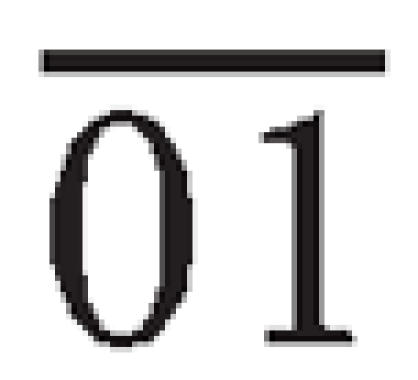
 ozone (95% posterior interval).

	Without PM adjustment	With adjustment by PM as specified by the row heading	No. of communities
All data	0.32 (0.17 to 0.46)	NA	98
With corresponding PM_10_ data	0.29 (0.03 to 0.55)	0.21 (−0.06 to 0.47)	93
With corresponding PM_2.5_ data	0.22 (−0.22 to 0.65)	0.21 (−0.22 to 0.64)	62

NA, not applicable.
